# Regulation, genomics, and clinical characteristics of cuproptosis regulators in pan-cancer

**DOI:** 10.3389/fonc.2022.934076

**Published:** 2022-10-27

**Authors:** Cankun Zhou, Chaomei Li, Yuhua Zheng, Xiaobin Huang

**Affiliations:** ^1^ Department of Gynecology, Southern Medical University Affiliated Maternal and Child Health Hospital of Foshan, Foshan, Guangdong, China; ^2^ Department of Maternity Centre, Southern Medical University Affiliated Maternal and Child Health Hospital of Foshan, Foshan, Guangdong, China

**Keywords:** cuproptosis, pan-cancer, genomics, tumor microenvironment, immune

## Abstract

**Background:**

Cuproptosis, a copper-dependent controlled cell death, is a novel form of cell death that differs from known cell death mechanisms; however, its overall regulation in cancer remains elusive.

**Methods:**

Multiple open-source bioinformatic platforms were used to comprehensively elucidate the expression levels, prognostic efficiency, potential biological functions, genomic and epigenetic characteristics, immune microenvironment, and drug sensitivity of cuproptosis regulators (ATP7A, ATP7B, DLAT, DLD, FDX1, GLS, LIAS, LIPT1, MTF1, NLRP3, PDHA1, PDHB, and SLC31A1) in pan-cancer.

**Results:**

Cuproptosis-related genes (CRGs) were upregulated in most cancers tested. In KIRC, KIRP, LGG, MESO, and PCPG, most highly expressed CRGs predicted a better prognosis but poorer prognosis in patients with ACC, LIHC, and UCEC. Pathway analysis confirmed that cuproptosis regulators were associated with the metabolism-related pathways. The expression of MTF1, NLRP3, and SLC31A1 was positively related with ImmuneScore, StromalScore, and ESTIMATEScore in almost all types of tumor, whereas ATP7B, DLAT, DLD, LIAS, PDHA1, and PDHB were significantly negatively correlated with the scores. In addition, CRGs were significantly correlated with RNA stemness score, DNA stemness score, microsatellite instability, and tumor mutational burden. The expression of ATP7A, ATP7B, LIAS, and DLAT was significantly positively correlated with the drug sensitivity of Docetaxel. ATP7A, LIAS, and FDX1 were significantly negatively correlated with the drug sensitivity of UNC0638, XMD13−2, YM201636, and KIN001−260.

**Conclusions:**

The altered genomic and clinical characteristics of cuproptosis regulators were comprehensively elucidated, providing a preliminary basis for understanding the functions of cuproptosis in pan-cancer.

## Introduction

Cancer is a malignant disease with a pathological manifestation of abnormal cell proliferation accompanied by dysregulated cell death and a disturbed inflammatory response. In addition, it has high morbidity and mortality. Cell death is mainly classified as accidental cell death and regulated cell death (RCD) (1). RCD is mediated by a set of pathways that play an important role in development and immune responses (2). To date, researchers have identified several RCD mechanisms; among which, apoptosis, pyroptosis, necroptosis, and ferroptosis are the four most widely investigated forms in recent years (3–6). A recent study on copper-related death (**cuproptosis**) published in *Science* on 17 March 2022 (7) is the first to suggest that cuproptosis is a novel form of cell death that is copper-dependent, regulated, and different from other known mechanisms of RCD.

Copper ions directly bind to lipoylated components in the tricarboxylic acid (TCA) cycle, leading to abnormal aggregation of lipoylated protein and the loss of iron–sulfur cluster proteins, thereby triggering proteotoxic stress responses and eventually mediating cell death. Copper levels are significantly altered in the serum and tumor tissue of patients in various cancers, including breast (8), thyroid (9), lung (10), colorectal (11, 12), oral cavity (13), prostate (14), and gallbladder (15) cancers. Growing evidence suggests that copper promotes angiogenesis, which is essential for tumor progression and metastasis development. This phenomenon is related to the fact that copper can activate angiogenesis-related factors, such as vascular endothelial growth factor, fibroblast growth factor 1, and angiopoietin (16, 17). Some studies have reported that high levels of copper can activate the function of Antioxidant 1 copper chaperone (ATOX1), increase the production of reactive oxygen species (ROS), and further enhance cell proliferation (18, 19). Immune escape of cancer cells is also an important mechanism of tumorigenesis, and studies have confirmed that copper in cancer cells can promote immune escape by overexpressing regulatory programmed death-ligand 1 (PD-L1) to protect the cells from tumor immune attack (20). Numerous studies have demonstrated that copper ion carriers increase the intracellular (especially mitochondrial) copper levels, which, in turn, increases ROS levels, eventually making cancer cells more susceptible to oxidative stress and leading to the development of cuproptosis (16, 17, 21). Therefore, copper metabolism plays a key role in the development of cancer and has emerged as a promising target for inhibiting cancer development. However, the complex relationship between cuproptosis-regulated genes and tumorigenesis requires further in-depth analysis.

To gain insights into the mechanisms of cuproptosis-related genes (CRGs) in cancer, we comprehensively analyzed the transcriptomic, clinical, epigenomic, and immunological characteristics of 33 human cancers using The Cancer Genome Atlas Program (TCGA) data. Significant differences were found in mRNA expression, prognostic efficiency, epigenetic characteristics, and tumor immune microenvironment among CRGs, and they were enriched in multiple metabolic pathways, providing a rich resource for understanding cuproptosis biology.

## Method

### Extraction of pan-cancer data and analysis of gene expression

On the basis of genome-wide CRISPR-Cas9 loss-of-function screening, Tsvetkov et al. found 10 regulatory genes specifically related to the cuproptosis metabolic pathway (7), including seven positively regulated genes [namely, ferredoxin 1 (FDX1), lipoic acid synthase (LIAS), lipoyltransferase-1 (LIPT1), dihydrolipoamide dehydrogenase (DLD), dihydrolipoamide S-acetyltransferase (DLAT), pyruvate dehydrogenase E1-alpha (PDHA1), and pyruvate dehydrogenase beta (PDHB)] and two negatively regulated genes [namely, metal transcription factor 1 (MTF) and glutaminase (GLS)]. Previous studies have also found that ATPase copper transporting alpha (ATP7A), ATPase copper transporting Beta (ATP7B), NLR family pyrin domain containing 3 (NLRP3), and solute carrier family 31 member 1 (SLC31A1) are closely associated with the cuproptosis metabolic process (12, 22–24).

The Xena Functional Genomics Explorer database (Xena, https://xena.ucsc.edu/) (25) was used to extract normal tissues in the Genotype-Tissue Expression (GTEx) database and TCGA pan-cancer data, including transcription expression data, clinical data, immunological subtypes, and stemness scores {based on mRNA [RNA stemness scores (RNAss)] and DNA [DNA stemness scores (DNAss)] methylation}. TCGA pan-cancer data includes 33 cancer types, namely, adrenocortical carcinoma (ACC), bladder urothelial carcinoma (BLCA), breast invasive carcinoma (BRCA), cervical squamous cell carcinoma and endocervical adenocarcinoma (CESC), cholangiocarcinoma (CHOL), colon adenocarcinoma (COAD), lymphoid neoplasm diffuse large B-cell lymphoma (DLBC), esophageal carcinoma (ESCA), glioblastoma multiforme (GBM), head and neck squamous cell carcinoma (HNSC), kidney chromophobe (KICH), kidney renal clear cell carcinoma (KIRC), kidney renal papillary cell carcinoma (KIRP), acute myeloid leukaemia (LAML), brain lower-grade glioma (LGG), lung squamous cell carcinoma (LUSC), liver hepatocellular carcinoma (LIHC), lung adenocarcinoma (LUAD), mesothelioma (MESO), ovarian serous cystadenocarcinoma (OV), prostate adenocarcinoma (PRAD), pancreatic adenocarcinoma (PAAD), pheochromocytoma and paraganglioma (PCPG), rectum adenocarcinoma (READ), stomach adenocarcinoma (STAD), sarcoma (SARC), skin cutaneous melanoma (SKCM), testicular germ cell tumor (TGCT), thyroid carcinoma (THCA), thymoma (THYM), uterine corpus endometrial carcinoma (UCEC), uveal melanoma (UVM), and uterine carcinosarcoma (UCS). Tumor data without healthy tissue samples were removed, including MESO and UVM. Differential gene expression analysis between tumor and paracancerous tissues was performed using a linear mixed-effects model. The expression profile of CRGs in 1,062 cancer cell lines was obtained from Cancer Cell Line Encyclopedia (CCLE, http://www.broadinstitute.org/ccle/home) (26). The expression of CRGs in normal tissues in the GTEx database (27) was visualized on a heatmap.

### Survival analysis of cuproptosis-related genes

The survival data of 33 TCGA cancer samples were extracted, including overall survival (OS), disease-specific survival (DSS), disease-free interval (DFI), and progression-free interval (PFI) data. On the basis of a Cox proportional hazards regression model, the “survival” R package was used to investigate whether the expression of CRGs was associated with the survival of patients, and the “ggplot2” R package was used to draw a diagonal heatmap for visualizing results. Independent variables with a hazard ratio (HR) of >1 and <1 were referred to as risk and protective factors, respectively, with a threshold specified as a P-value of <0.05.

### Functional enrichment analysis

Gene Set Cancer Analysis (GSCALite, http://bioinfo.life.hust.edu.cn/GSCA/) is a user-friendly comprehensive cancer analysis database that integrates multigene, mutation, and drug sensitivity analyses spanning 33 cancer types in TCGA and Genomics of Drug Sensitivity in Cancer (GDSC) data (28). The tumor pathway activity module contains the activities of 10 cancer pathways, namely, apoptosis, cell cycle, DNA damage, epithelial–mesenchymal transition (EMT), hormone androgen receptor (AR), hormone estrogen receptor (ER), phosphatidylinositol-4,5-bisphosphate-3-kinase (PI3K)/protein kinase B (AKT), RAS/mitogen-activated protein kinase (MAPK), receptor tyrosine kinase (RTK), and tuberous sclerosis 1 protein (TSC)/mechanistic target of rapamycin (mTOR) pathways. The expression of CRGs was analyzed in relation to the activation or inhibition of the abovementioned oncogenic pathways. A protein–protein interaction (PPI) network of CRGs was constructed using the GeneMANIA platform (29) (http://genemania.org/), and functional enrichment analyses were performed to further understand the function of these genes in pan-cancer, including Gene Ontology (GO) and Kyoto Encyclopedia of Genes and Genomes (KEGG) pathway analyses.

### Genetic alteration

cBioPortal platform (https://www.cbioportal.org/) can be used to study genetic alteration characteristics (30). cBioPortal precisely presented the details of all forms of the genetic alterations of structural variants, mutations, amplifications, deep deletions, and copy number alterations with the CRGs in pancancer by the OncoPrint module.

### Copy number variation analysis

The copy number variation (CNV) module of the GSCALite platform contained CNV data of 11,495 samples from TCGA database, including homozygous and heterozygous deletion, diploid (none), and heterozygous and homozygous amplification, downloaded from TCGA database and processed by GISTIC2.0. The four CNV types of CRGs in pan-cancer were summarized using a pie chart, and Spearman correlation analysis was performed to analyze the correlation between the mRNA expression of CRGs and CNVs. Finally, survival differences between mutants and WT were analyzed using a Cox proportional hazards regression model and log-rank test.

### Methylation analysis

The genome is considered to be affected by one conventional epigenetic alteration, DNA methylation. Using the methylation module of the GSCALite platform, the methylation data of 10,129 samples spanning 33 cancer types were extracted from the National Cancer Institute (NCI) Genomic Data Commons. Data on 14 cancer types were extracted, with paired healthy tissue data, and differences in methylation between cancerous and healthy tissue samples of these 14 cancer types (including BLCA, BRCA, COAD, ESCA, HNSC, KIRC, KIRP, LIHC, LUAD, LUSC, PAAD, PRAD, THCA, and UCEC) were analyzed using the Student’s t-test. Spearman correlation analysis was performed to analyze the correlation between the mRNA expression of CRGs and methylation. Finally, survival differences between the high- and low-methylation groups were compared using a Cox proportional hazards regression model and log-rank tests.

### Tumor microenvironment and immune subtype analysis

ESTIMATE is a tool used for predicting tumor purity (31). The expression data of CRGs were used to predict the number of infiltrating stromal/immune cells in tumor tissues and subsequently calculate the immune and stromal scores. Spearman correlation analysis was used to analyze the correlation between the genes and scores. The correlation between the expression of CRGs and six types of immune cells (B cells, CD4+ T cells, CD8+ T cells, neutrophils, macrophages, and dendritic cells) in 32 human cancers was analyzed using the Tumor IMmune Estimation Resource 2.0 website (Timer2.0, http://timer.cistrome.org/) (32) and visualized on heatmaps generated using the “ggplot2” R package. Thorsson et al. conducted an extensive immunogenomic analysis of 33 cancers in TCGA database and identified six immune subtypes, including C1 (wound healing), C2 (Interferon (IFN)-gamma dominant), C3 (inflammatory), C4 (lymphocyte depleted), C5 (immunologically quiet), and C6 (Transforming growth factor beta (TGF-b) dominant) (33). The pan-cancer immune subtypes were obtained from the Xena database, and the correlation between the expression of CRGs and the immune subtypes was assessed using ANOVA.

### Correlation between cuproptosis-related gene expression and tumor mutational burden, microsatellite instability, and stemness scores

Tumor mutational burden (TMB) and microsatellite instability (MSI) are important biomarkers of the tumor microenvironment (TME) (34, 35). Spearman correlation analysis was used to analyze the correlation between the expression of CRGs and the abovementioned TME biomarkers. In addition, RNAss and DNAss can be used to assess the stem cell–like characteristics of tumors (36). Spearman correlation analysis was used to analyze the correlation between the expression of CRGs and RNAss/DNAss. Heatmaps were plotted using the “ggplot2” R package, and differences with a P-value of <0.05 were considered statistically significant.

### Drug sensitivity analysis

The GDSC project collected 265 small molecules (37) and analyzed the correlation between the expression of CRGs and drug sensitivity according to the method used by Rees et al. (38). Spearman correlation was used to assess the correlation between gene expression and small-molecule drugs, in which a positive correlation indicated that samples with high gene expression were resistant to the drug. The NCI-60 cell line is the most widely used population of cancer cells for anti-cancer drug testing, and its gene expression and drug sensitivity data [including multiple Food and Drug Administration (FDA)–approved drugs and drug molecules in clinical trials] is available in the CellMiner database (https://discover.nci.nih.gov/cellminer/home.do) (39). Spearman correlation analysis was used to assess the correlation between the expression of CRGs and sensitivity to FDA-approved drugs.

## Result

### Expression levels of cuproptosis-related genes in pan-cancer

**Figure 1:**
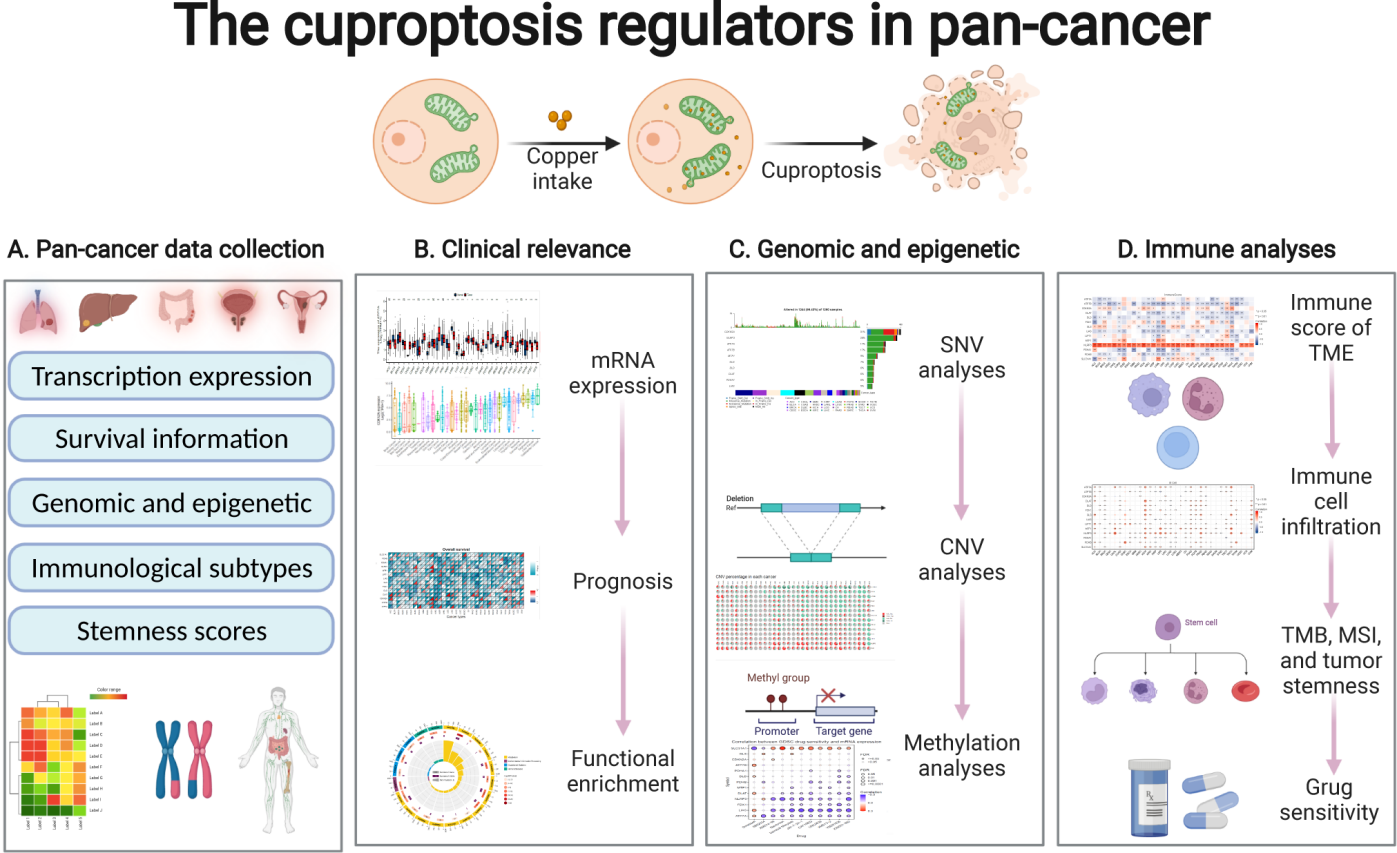
General overview of the study.

**Figure 2:**
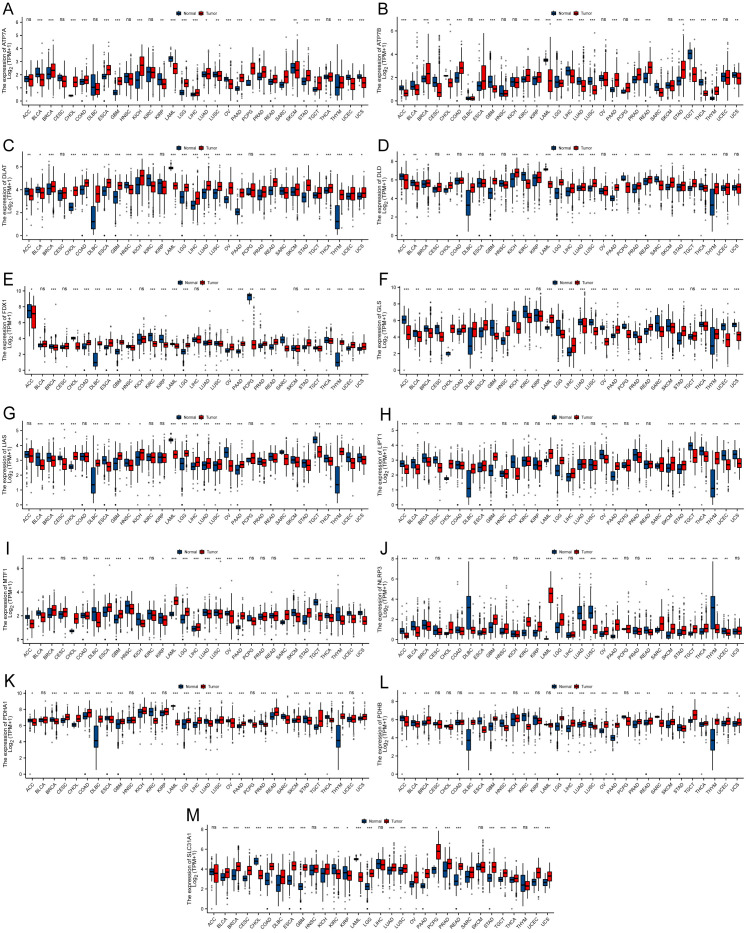
Boxplot demonstrating the differential expression of cuproptosis-related genes between cancerous and adjacent healthy tissues. The blue color indicates healthy tissues, and the red color indicates cancerous tissues. **(A)** ATP7A. **(B)** ATP7B. **(C)** DLAT. **(D)** DLD. **(E)** FDX1. **(F)** GLS. **(G)** LIAS. **(H)** LIPT1. **(I)** MTF1. **(J)** NLRP3. **(K)** PDHA1. **(L)** PDHB. **(M)** SLC31A1 (*P < 0.05; **P < 0.01; ***P < 0.001) No significance (ns).

**Figure 1** illustrates the workflow of the study. Studies have reported that the expression of CRGs is significantly altered in various cancers. In this study, among the 33 TCGA cancer types, excluding cancer data without healthy tissue samples, 31 cancers were eventually included for differential expression analysis. Significant differential expression of almost all CRGs was observed among different cancer types (P < 0.05) and as upregulated in the majority of cancers tested (**Figure 2**). Furthermore, the expression of CRGs was observed in various healthy tissues in the GTEx dataset. As shown in **Supplementary Figure S1** the expression of DLD and FDX1 in the adrenal gland; ATP7A in the bladder; GLS in blood vessels; PDHA1 in the heart; SLC31A1 in the liver; DLAT and PDHB in muscle; NLRP3 in the spleen; and ATP7B, LIAS, LIPT1, and MTF1 in the testis was significantly upregulated. In addition, the expression of these genes in cancer cell lines was further analyzed using the CCLE dataset (**Supplementary Figure S2**). The expression of ATP7A, ATP7B, DLAT, DLD, FDX1, GLS, LIAS, LIPT1, MTF1, NLRP3, PDHA1, PDHB, and SLC31A1 was higher in brain cancer, colon/colorectal cancer, rhabdoid, rhabdoid, bone cancer, kidney cancer, eye cancer, teratoma, thyroid cancer, leukemia, teratoma, rhabdoid, and fibroblast cell lines, respectively, than in other cancer cell lines.

### Prognostic value of cuproptosis-related genes in pan-cancer

**Figure 3:**
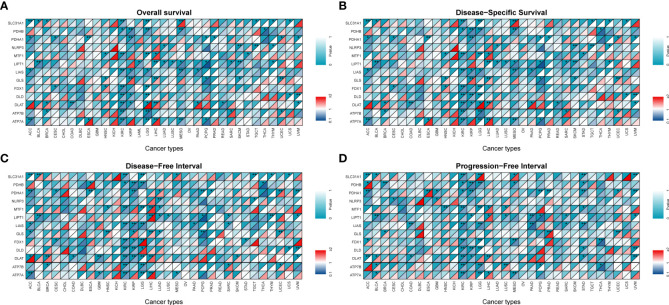
Survival analysis and genetic alterations of cuproptosis-related genes. Diagonal split heatmap demonstrating that the expression of cuproptosis-related genes correlates with overall survival **(A)**, disease-specific survival **(B)**, disease-free interval **(C)**, and progression-free interval **(D)** in patients with different cancer types using univariate Cox proportional hazard regression models. The upper and lower triangles indicate significance and hazard ratio (HR), respectively. Independent variables with HR of <1 are referred to as protective factors (dark blue area in the bottom right corner), and those with HR of >1 are referred to as risk factors (colored area in the bottom right corner except for dark red), with a threshold specified as a P-value of <0.05 (*P < 0.05; **P < 0.01).

A univariate Cox HR regression model was established in TCGA to analyzed four survival endpoints (OS, DSS, DFI, and PFI) to determine the prognostic value of CRGs. Survival analysis showed that most high CRG expression predicted a better prognosis in KIRC, KIRP, LGG, MESO, and PCPG, which both played a protective role. However, most higher expression levels of CRGs were associated with poorer survival in patients with ACC, LIHC, and UCEC (**Figure 3**, P < 0.05). Detailed results are provided in **Table S1**. These results suggest that dysregulated expression of CRGs is associated with tumor prognosis.

### Functional enrichment analysis of cuproptosis-related genes in pan-cancer

**Figure 4:**
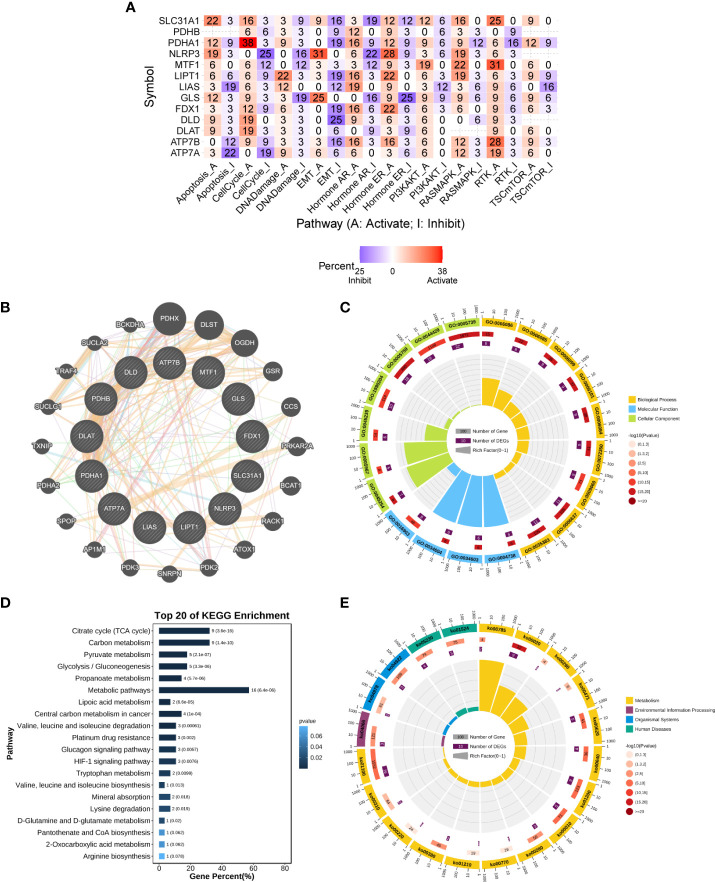
Enrichment analysis of cuproptosis-related genes. **(A)** Combined activity of cancer-related pathways in pan-cancer (GSCALite). **(B)** PPI network of cuproptosis-related genes (GeneMANIA). **(C)** GO enrichment analysis. **(D, E)** KEGG enrichment analysis. The numbers outside the circles are a scale of the number of genes. The first circle is the GO id (or KEGG id) label. The second circle shows the total number of genes in each ID classification in GO or KEGG and the P-value, the more genes the longer the bar; and the smaller the P-value, the redder the color. The third circle shows the number of input genes in each ID classification. The fourth circle shows the RichFactor value (the ratio of input genes to the total number of classified genes in this classification) for each classification, with each cell of the background auxiliary line indicating 0.1. GO, Gene Ontology; KEGG, Kyoto Encyclopedia of Genes and Genomes; PPI, protein–protein interaction.

We analyzed CRGs involved in well-known cancer-related signaling pathways (apoptosis, cell cycle, DNA damage response, EMT, hormone AR, hormone ER, PI3K/AKT, RAS/MAPK, RTK, and TSC/mTOR). In pan-cancer, CRGs were found to be closely associated with most well-known cancer-related pathways. Most CRGs tended to activate the apoptosis, cell cycle, PI3K-AKT, and RAS-MAPK pathways more than the inhibitory effects (**Figure 4A**).

The set of genes closely regulating cuproptosis was obtained through the PPI regulatory network (**Figure 4B**) and subsequently subjected to functional enrichment analysis. In the GO biological process (BP) category, these genes were found to be mainly involved in the acetyl-CoA biosynthetic and metabolic processes (GO:0006086/GO:0006085/GO:0071616/GO:0006084/GO:0006637), the TCA cycle (GO: 0006099), and TCA metabolism (GO:0072350). In the molecular function (MF) category, these genes were found to be mainly involved in pyruvate dehydrogenase activity (GO:0004738/GO:0034603/GO:0034604). In the cellular component (CC) category, these genes were found to be mainly involved in pyruvate dehydrogenase complex (GO:0045254) and mitochondrial part (GO:0005967/GO:0005759/GO:0044429/GO:0005739) (**Figure 4C** and **Table S2**). Furthermore, KEGG pathway analysis showed that these genes were enriched in multiple metabolic pathways, including lipoic acid metabolism, citrate cycle (TCA cycle), and glycolysis/gluconeogenesis. In addition, these genes were highly enriched in the HIF-1 signaling pathway, glucagon signaling pathway, and central carbon metabolism in cancer (**Figures 4D, E** and **Table S2**). These results suggest that CRGs play an important role in human cancers through metabolism-related pathways.

### Genomic mutation analysis of cuproptosis-related genes

**Figure 5:**
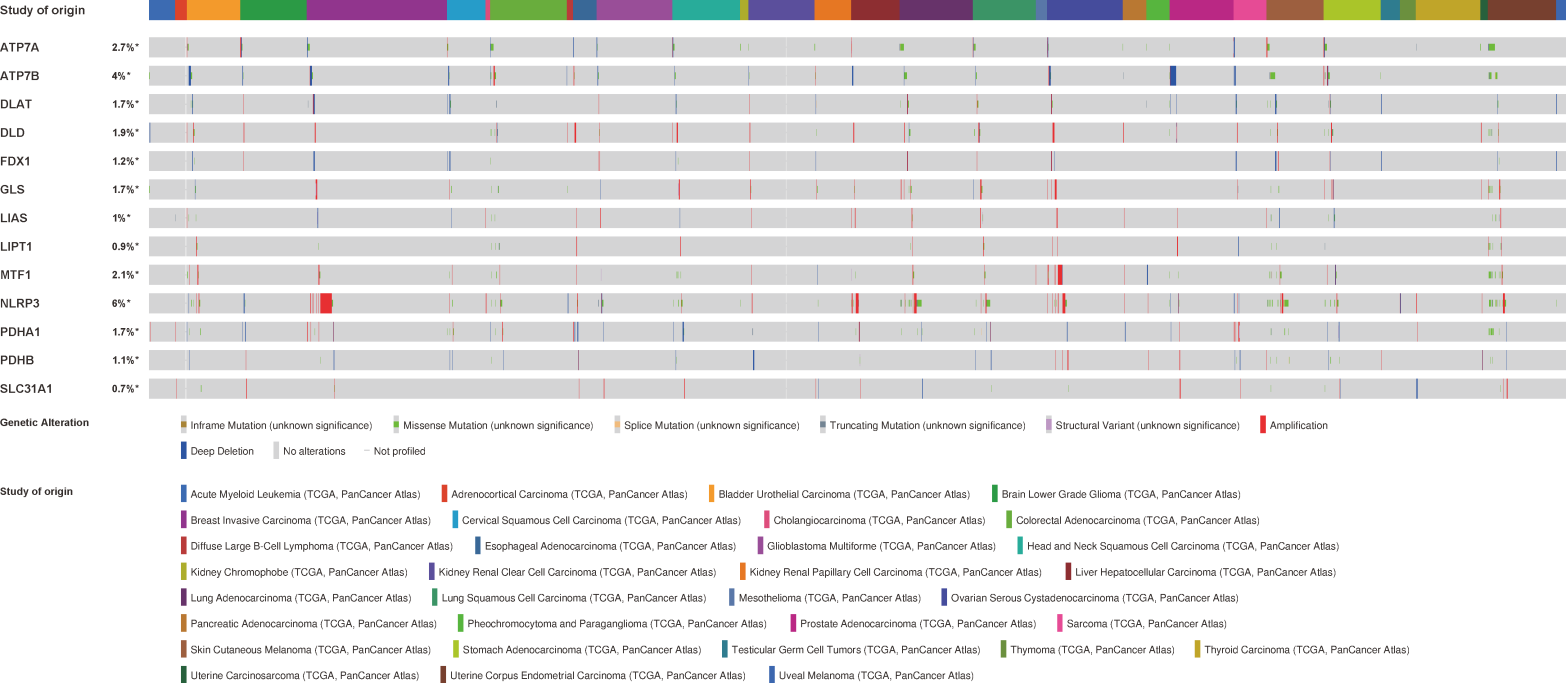
The genetic alteration characteristics of cuproptosis-related genes across different tumors (cBioPortal).

To assess the mutation of CRGs in pan-cancer, we conducted an in-depth study using the cBioPortal database and found that the mutation frequency of ATP7A, ATP7B, DLAT, DLD, FDX1, GLS, LIAS, LIPT1, MTF1, NLRP3, PDHA1, PDHB, and SLC31A1 was 2.7%, 4%, 1.7%, 1.9%, 1.2%, 1.7%, 1%, 0.9%, 2.1%, 6%, 1.7%, 1.1.%, 0.7%, respectively. Amplification was the most common type of gene variation (**Figure 5**).

**Figure 6:**
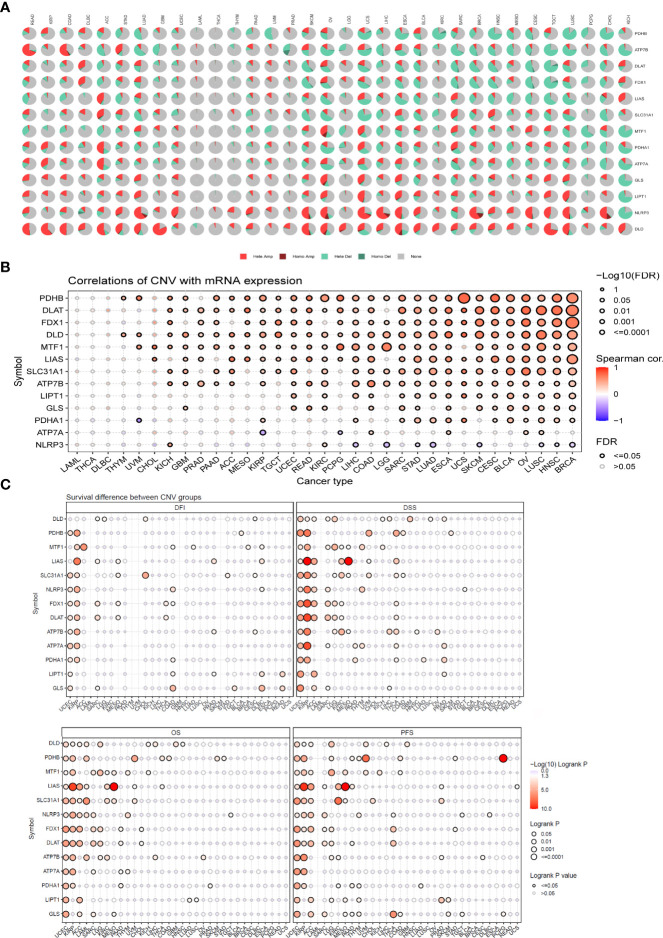
CNV analysis of cuproptosis-related genes (GSCALite). **(A)** Pie chart summarizing the CNVs of cuproptosis-related genes in pan-cancer, namely, heterozygous amplification (Hete Amp), homozygous amplification (Homo Amp), heterozygous deletion (Hete Del), homozygous deletion (Homo Del), and diploid (none). **(B)** The correlation between CNVs and mRNA expression was analyzed *via* Spearman correlation analysis. The blue dots represent negative correlations, and the red dots indicate positive correlations; the darker the color, the higher the correlation. The size of the dots represents statistical significance, with larger dots being more significant. **(C)** Differences in survival between the mutant and WT of each gene in the cuproptosis-related gene set. If the hazard ratio (HR) was >1, the CNV group was considered to have a higher risk of death; otherwise, the WT group was considered to have a higher risk of death. CNV, copy number variation.

The CNV pie plot demonstrated that the two main types of CNVs were heterozygous amplification (Hete Amp) and heterozygous deletion (Hete Del) (**Figure 6A**), and the proportion of CNVs per gene in each cancer is shown in **Table S3**. The correlation analysis showed that the mRNA expression of a vast majority of CRGs was positively correlated with CNVs (P < 0.05, **Figure 6B**). Prognostic analysis revealed that the risk of death was higher for most CRGs in the CNV (mutant) group than in the WT group (P < 0.05, **Figure 6C**). These results suggest that CNVs of CRGs mediate their aberrant expression, which may be closely related to the development and progression of cancer.

**Figure 7:**
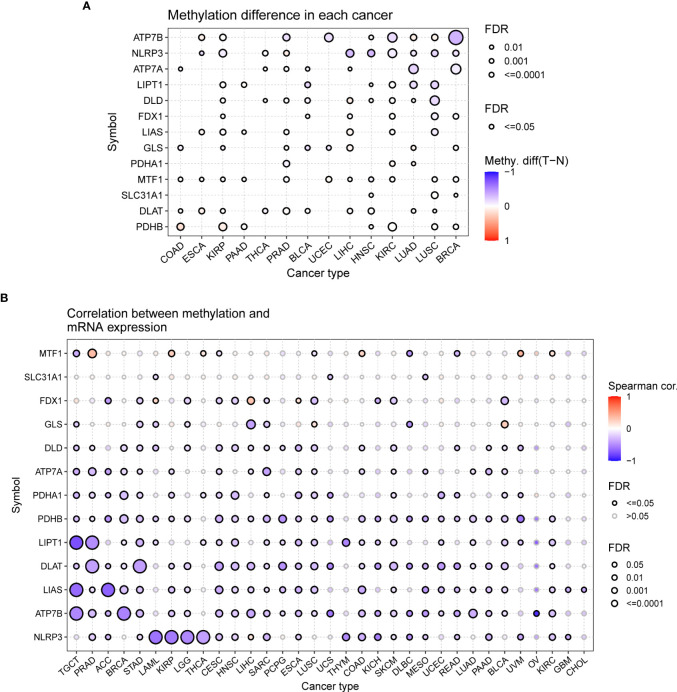
Methylation analysis of cuproptosis-related genes (GSCALite). **(A)** Methylation bubble plot showing differences in the methylation of cuproptosis-related genes between tumor and healthy tissue samples. The blue dots represent downregulation of methylation in tumor tissues compared with healthy tissues, and the red dots represent upregulation of methylation in tumor tissue compared with healthy tissues; the darker the color, the greater the difference. The size of the dots represents statistical significance, with larger dots being more significant. **(B)** The correlation between methylation and mRNA expression was analyzed *via* Spearman correlation analysis. The blue dots represent negative correlations, and the red dots indicate positive correlations; the darker the color, the higher the correlation. The size of the dots represents statistical significance, with larger dots being more significant.

DNA methylation, as one of the common epigenetic events, plays a key role in the diagnosis and treatment of tumors (40). In contrast to other CGRs, ATP7B, NLRP3, and ATP7A were distinctly hypomethylated in multiple cancers, such as BRCA, LUSC, and LUAD. PDHB was hypermethylated in COAD, KIRP, PAAD, HNSC, KIRC, LUSC, and BRCA (P < 0.05, **Figure 7A**). The differential methylation levels of CRGs may be due to differences in expression patterns between tumor and normal tissues. To test this conjecture, we further assessed the correlation between DNA methylation levels and mRNA expression. ATP7B and NLRP3 showed a negative correlation in most cancers (P < 0.05, **Figure 7B**). Prognostic analysis for OS, DSS, and PFS revealed that hypomethylation of DLAT, FDX1, MTF1, NLRP3, and PDHA1 was associated with low survival in most cancers, whereas hypermethylation of FDX1 and PDHB was associated with low survival in UVM(P < 0.05, **Supplementary Figure S3**).

### Cuproptosis-related gene expression is related to the tumor microenvironment and immune infiltrate subtypes in pan-cancer

**Figure 8:**
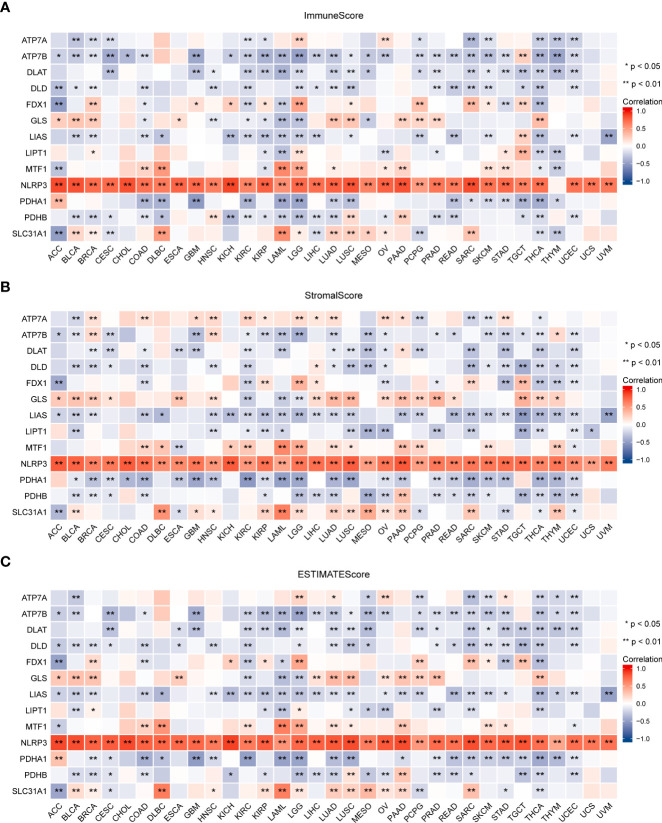
Association between the expression of cuproptosis-related genes and the tumor immune microenvironment in pan-cancer. Association between the expression of cuproptosis-related genes and immune scores **(A)**, stromal scores **(B)**, and ESTIMATE scores **(C)** (*P < 0.05; **P < 0.01). ESTIMATE, Estimation of Stromal and Immune cells in Malignant Tumor tissues using Expression data.

**Figure 9:**
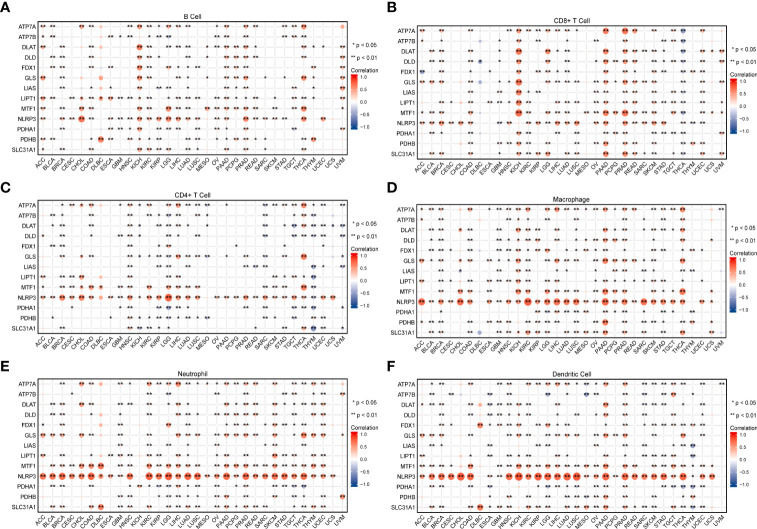
The relationship between the expression of cuproptosis-related genes and infiltrating levels of B cells **(A)**, CD8+ T cells **(B)**, CD4+ T cells **(C)**, macrophages **(D)**, neutrophils **(E)**, and dendritic cells **(F)** in pan-cancer was analyzed using the TIMER database (*P < 0.05; **P < 0.01).

Tumor tissue and the tumor immune microenvironment (TIME) are dependent on each other, and immune and inflammatory factors in the TIME play a key role in the immunotherapeutic response (41). In this study, TME analysis revealed that MTF1, NLRP3, and SLC31A1 were significantly positively correlated with TME scores (ImmuneScore, StromalScore, and ESTIMATEScore) in most human cancers, whereas ATP7B, DLAT, DLD, LIAS, PDHA1, and PDHB were significantly negatively correlated with the scores in most human cancers (**Figure 8**; details are provided in **Table S4**). In addition, CRGs were significantly differentially expressed in different immune subtypes (**Supplementary Figure S4**). ATP7B, DLAT, and PDHA1 were highly expressed in the C1 (wound healing) subtype; MTF1and SLC31A1 were highly expressed in the C2 (IFN-gamma dominant) subtype; ATP7A, FDX1, GLS, and LIPT1 were highly expressed in the C3 (inflammatory) subtype; DLD was highly expressed in the C4 (lymphocyte depleted) subtype, whereas LIAS and PDHB were highly expressed in the C5 (immunologically quiet) subtype; NLRP3 was highly expressed in the C6 (TGF-b dominant). Furthermore, a significant positive correlation was observed between the expression of CRGs and the infiltration of B cells, CD8+ T cells, macrophages, neutrophils, and dendritic cells across 32 cancer types (**Figure 9**; details are provided in **Table S5**). These results suggest that dysregulated expression of the cuproptosis gene family may mediate disturbances in the TIME, which, in turn, promotes immunosuppression.

### Cuproptosis-related gene expression is related to TMB, MSI, and stemness scores in pan-cancer

**Figure 10:**
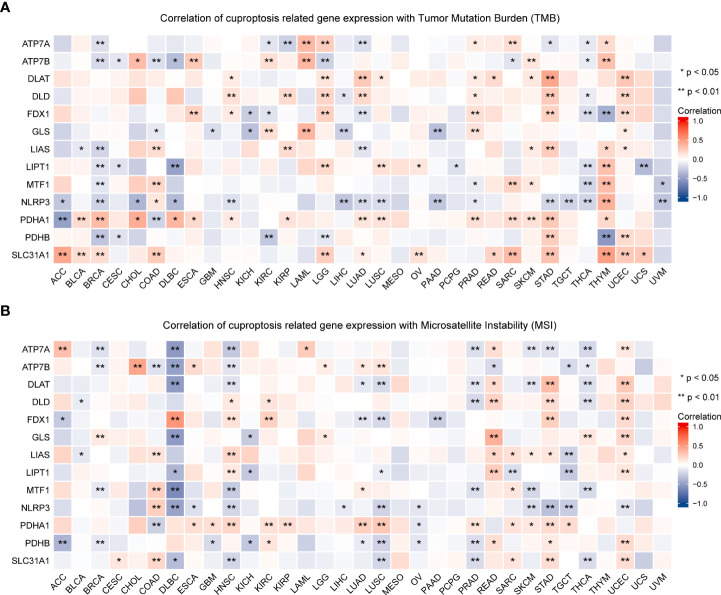
Association of the expression of cuproptosis-related genes with TMB **(A)** and MSI **(B)** in pan-cancer (*P < 0.05; **P < 0.01). TMB, tumor mutational burden; MSI, microsatellite instability.

**Figure 11:**
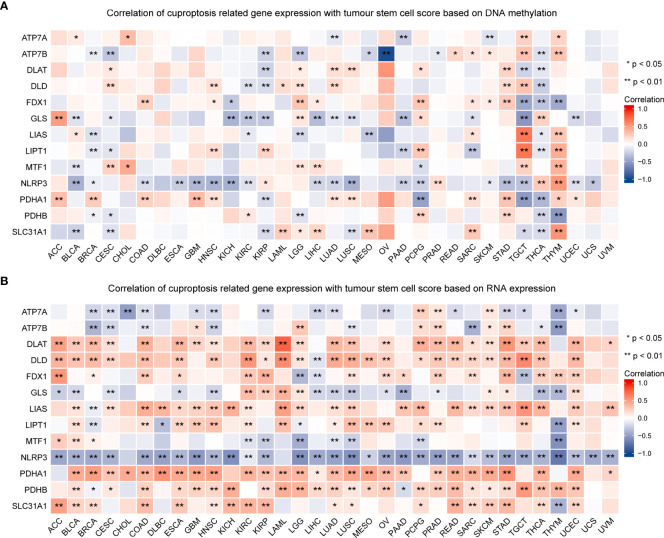
Association of the expression of cuproptosis-related genes with tumor stemness in pan-cancer. The correlation between the expression of cuproptosis-related genes and tumor stemness scores based on DNA methylation **(A)** and RNA expression **(B)** (*P < 0.05; **P < 0.01).

TMB and MSI in the TME are effective prognostic biomarkers and indicators of immunotherapeutic response in tumors (42–44). To examine the role of CRGs in anti-tumor immunity in the TME, we analyzed the correlation between the expression of these genes and TMB and MSI and found that CRG expression was significantly correlated with TMB and MSI in most tumors. For example, FDX1 expression was significantly positively correlated with TMB and MSI in HNSC, STAD, and UCEC but was significantly negatively correlated with TMB and MSI in LUAD (P < 0.05; **Figure 10**, **Table S6**). These findings suggest the biological relevance of CGRs in PD1/PD-L1 therapy. In addition, the expression of CRGs was found to be associated with tumor stemness in pan-cancer (**Figure 11**, **Table S6**). The results showed that the expression of CRGs was significantly positively correlated with RNAss in a vast majority of tumors and that of ATP7A and NLRP3 was negatively correlated with RNAss. The expression of LIAS and LIPT1 was significantly positively correlated with DNAss, whereas that of FDX1, GLS, NLRP3, and PDHA1 was significantly negatively correlated with DNAss, with a large correlation coefficient.

### Drug sensitivity analysis of cuproptosis-related genes

**Figure 12:**
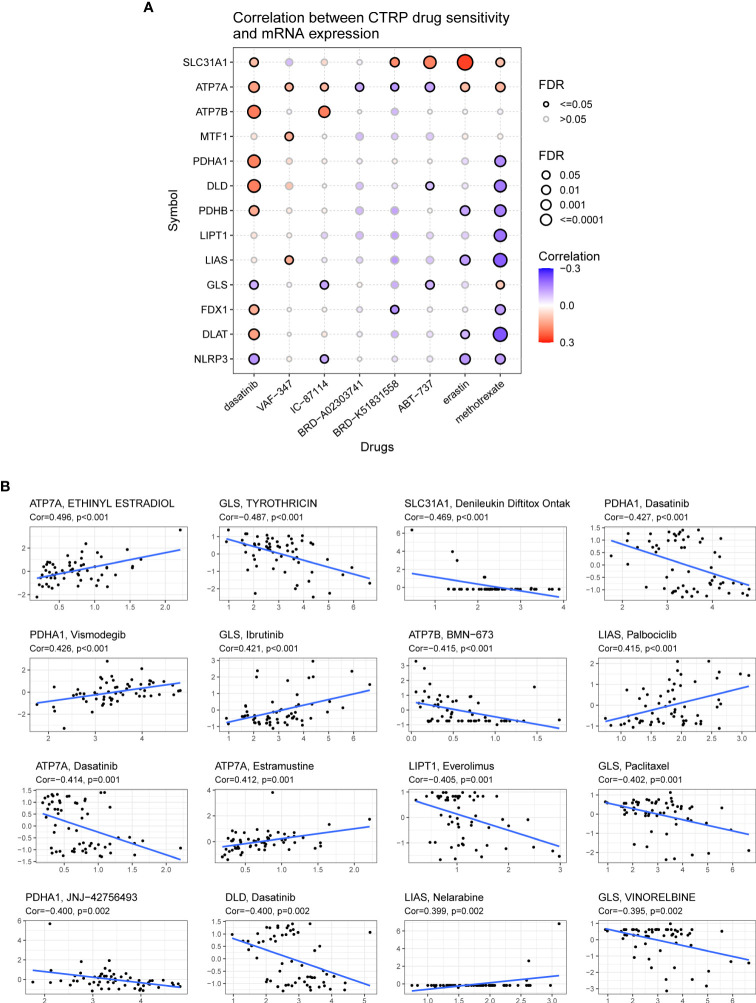
Association of the expression of cuproptosis-related genes with drug sensitivity. **(A)** Spearman correlation represents drug-related gene expression (GDSC). A positive correlation indicates that the gene is highly resistant to drugs. **(B)** The associations of cuproptosis-related genes expression and drug sensitivity based on CellMiner dataset, where the x- and y-axes represent gene expression and drug sensitivity.

The correlation between the expression of CRGs and drug sensitivity was analyzed in different human cancer cell lines using the GDSC and CellMiner™ databases (**Figure 12**). Spearman correlation analysis showed that the half maximal inhibitory concentration (IC-50) values of Docetaxel were positively correlated with the expression of ATP7A, ATP7B, LIAS, and DLAT, whereas those of UNC0638, XMD13−2, YM201636, and KIN001−260 were negatively correlated with the expression of ATP7A, LIAS, and FDX1 (**Figure 12A**). Another database analysis showed that ATP7A expression was positively correlated with the drug sensitivity of ETHINYL ESTRADIOL and Estramustine but was negatively correlated with the drug sensitivity of Dasatinib and JNJ−42756493; GLS expression was positively correlated with the drug sensitivity of Ibrutinib but was negatively correlated with the drug sensitivity of TYROTHRICIN, Paclitaxel, and VINORELBINE (**Figure 12B**).

## Discussion

Elevated copper ion levels in cancer tissues can promote angiogenesis and immune escape, which, in turn, promotes tumor growth and metastasis (16, 20). In many clinical trials, different classes of copper ion carriers have been used for the treatment of cancer through mitochondrial oxidative stress and cuproptosis (44, 45). However, the field is in the early stages of development. Therefore, it is necessary to investigate the role of cuproptosis regulators in tumor development and to identify potential targets for clinical treatment. In this study, bioinformatic multiomic analyses provided a comprehensive understanding of the role of cuproptosis regulators in human cancers and propagated the search for new therapeutic targets.

In this study, we investigated the differential expression of CRGs (ATP7A, ATP7B, DLAT, DLD, FDX1, GLS, LIAS, LIPT1, MTF1, NLRP3, PDHA1, PDHB, and SLC31A1) in cancer versus healthy tissues and healthy tissues versus cancer cell lines using the transcriptomic data derived from 33 different tumors, healthy tissues, and cancer cell lines in TCGA, GTEx, and CCLE datasets, respectively. Significant heterogeneity was found in the expression of these genes within tumors, between tumors and among cancer cell lines as well as between tumors in terms of prognosis, thus necessitating the study of each cuproptosis family member. A growing number of pan-cancer analyses have shown that genetic mutations are associated with tumorigenesis and progression (46). Our findings showed that the high frequency of copy number alterations in CRGs, mainly Hete Amp and Hete Del, and their significant positive correlation with gene expression accompanied by a higher risk of death suggest that copy number alterations may contribute to cancer development and progression by affecting gene expression. Moreover, compared with other CGRs, ATP7B, NLRP3, and ATP7A are significantly hypomethylated in various cancers, such as BRCA, LUSC, and LUAD. In addition, PDHB is hypermethylated in COAD, KIRP, PAAD, HNSC, KIRC, LUSC, and BRCA. Therefore, we hypothesized that hypomethylation induced the transcriptional activation of DLAT, FDX1, and PDHA1 genes, leading to cell death.

Functional pathway analysis revealed that CRGs were closely associated with most well-known cancer-related pathways. It is now well established that copper intake is critical for the activity of MEK1 and MEK2 in the RAS/MAPK signaling pathway and for activating the phosphorylation of ERK1 and ERK2, and the activation of this pathway is a key factor in promoting tumor growth (47). Among the cuproptosis regulators analyzed in this study, most CRGs tended to activate the AS-MAPK pathways more than the inhibitory effects. In addition, functional enrichment analysis of the cuproptosis regulator PPI verified that cuproptosis regulators and their co-expressed genes are involved in various metabolic processes, especially TCA metabolism. Previous studies have found that copper, as a cofactor in the catalysis of most essential enzymes in the body, is frequently involved in energy production, oxygen transport, and cellular metabolism (48–50). Cancer cells have higher copper requirements than normal cells. Through the direct binding of copper to the fatty acylated component of TCA, some cancers abnormally express many lipoylated mitochondrial proteins and exhibit high-intensity respiration, which eventually leads to cell death (7). Therefore, cuproptosis regulators may constitute a network of interactions in cancer-related signaling pathways that promote the development of cuproptosis.

The TME plays a crucial role in stimulating tumor cell heterogeneity, multidrug resistance, cancer progression, and metastasis (51). In the TME and immune cell infiltration correlation analysis, the expression of CRGs was found to be significantly correlated with immune scores and immune cell infiltration levels in some tumors, with a significant positive correlation between NLRP3 and TME in particular. Activation of NLRP3 inflammasome has been shown to affect inflammatory cell death, to mediate the secretion of pro-inflammatory cytokines, and to influence anti-tumor immunity (52). In a study, copper-treated mice exhibited ROS production and changes in the mitochondrial transmembrane potential, mainly leading to immunotoxicity in the form of reduced CD4+ T-cell populations and increased or proliferating CD4+ T-cell populations (53). In another study, copper chelators significantly increased the number of tumor-infiltrating natural killer cells, delayed tumor growth, and improved survival in mice by decreasing intracellular copper concentrations (20). In addition, copper chelators (CuNG) modulate the transition of tumor-associated macrophages from an immunosuppressed to a pro-immunogenic state (54). In this study, FDX1 expression was significantly positively correlated with immune scores and tumor immune cell infiltration in several human cancers. Moreover, for the regulation of cuproptosis, FDX1 is the key positive regulator of copper ion carrier–induced cell death, and high expression of FDX1 is accompanied by high immune cell infiltration, potentially leading to cell death through immunosuppressive effects. Overall, the interaction between the CRG family and immune cells in tumors may provide new perspectives for the development of more effective therapeutic strategies.

During cancer progression, tumor cells gradually lose their differentiated phenotype and acquire progenitor and stem cell–like features, and stem cell indices are associated with active BPs of cancer stem cells and tumor dedifferentiation (55). In this study, the expression of CRGs was significantly correlated with DNAss and RNAss, with RNAss being significantly positively correlated with corresponding gene expression in most tumors. However, there were contradictory results of positive and negative correlations between the expression of CRGs and RNAss and DNAss of individual tumors. For example, in TCHA and THCA, FDX1 expression showed a significant positive correlation with RNAss and a significant negative correlation with DNAss. These contradictory results suggest that combining DNAss and RNAss can identify different tumor features or tumor cell populations characterized by different stemness.

Another important finding of this study is the association between CRG expression and TMB and MSI in some cancer types. It is now well established that TMB and MSI can help to predict the response of patients to various drugs, particularly immune checkpoint inhibitors (35, 56). In addition, high TMB and MSI are associated with a good response to immune checkpoint inhibitors (57, 58). In this study, FDX1 expression was significantly positively correlated with TMB and MSI in HNSC, STAD, and UCEC, and DLAT expression was significantly positively correlated with TMB and MSI in READ, STAD, and UCEC, suggesting that these genes may be potential indicators of drug response. In addition, genes such as ATP7A, ATP7B, FDX1, GLS, and PDHA1 have been mined for identifying potential drug targets for drug sensitivity analysis. Tsvetkov et al. found that FDX1 is a direct target of elesclomol and both act in correlation, with increased ROS production owing to increased copper uptake, eventually leading to copper-dependent cell death in cancer cells (59). FDX1 has been reported to enhance the copper-dependent cell death induced by elesclomol, providing a new idea to improve the efficacy of cancer-targeted drugs (59). Therefore, detecting the expression of CRGs in patients with cancer is relevant for guiding the selection of clinical drugs. However, further studies are required to verify these results and clarify the potential mechanisms underlying drug regulation of CRGs.

Although this is the first study to multidimensionally analyzed CRGs across multiple cancer types, it has some limitations. First, all results are based on public databases and have not been validated using other independent databases. Second, the underlying mechanisms behind the bioinformatic analysis have not been explored through molecular and animal experiments. Therefore, the specific biological role and mechanism of CRGs warrant further experimental validation.

The study provides a comprehensive analysis of CRGs in pan-cancer. Upregulation of CRG expression was observed in most cancers compared with normal tissues. Survival analysis confirmed that most highly expressed CRGs had a better prognosis in KIRC, KIRP, LGG, MESO, and PCPG and a worse prognosis in patients with ACC, LIHC, and UCEC. The pathway results suggest that CRGs are mainly involved in tumor metabolism-related signaling pathways. In addition, CRGs correlated with the level of immune cell infiltration, TMB, MSI, and tumor stemness score, with NLRP3 being more strongly correlated. In conclusion, these findings may provide new insights into CRGs as potential therapeutic targets in pan-cancer.

## Data availability statement

Publicly available datasets were analyzed in this study. The datasets presented in this study can be found in online repositories. The name and login number of the repository/repository are as follows: TCGA and GTEx form Xena (https://xena.ucsc.edu/), CCLE (http://www.broadinstitute.org/ccle/home), GSCA (http://bioinfo.life.hust.edu.cn/GSCA/), Timer2.0 (http://timer.cistrome.org/), GDSC (https://www.cancerrxgene.org/), TISIDB (http://cis.hku.hk/TISIDB/) and CellMiner (https://discover.nci.nih.gov/cellminer/home.do).

## Author contributions

CZ and YZ performed data analysis work and aided in writing the manuscript. CZ and XH designed the study and assisted in writing the manuscript. CL edited the manuscript. All authors read and approved the final manuscript.

## Funding

This work was supported by grants from the Foshan Transnatural Cavity Surgery Development and Innovation Engineering Technology Research Center (2020001003507) and 2020 Foshan City self-financing science and technology projects (2020001005656).

## Acknowledgments

We thank Bullet Edits Limited for the linguistic editing and proofreading of the manuscript.

## Conflict of interest

The authors declare that the research was conducted in the absence of any commercial or financial relationships that could be construed as a potential conflict of interest.

## Publisher’s note

All claims expressed in this article are solely those of the authors and do not necessarily represent those of their affiliated organizations, or those of the publisher, the editors and the reviewers. Any product that may be evaluated in this article, or claim that may be made by its manufacturer, is not guaranteed or endorsed by the publisher.

## Supplementary material

Supplementary Table 1 Survival analysis and genetic alterations of cuproptosis-related genes in patients with different cancer types using univariate Cox proportional hazard regression models.

Supplementary Table 2 Enrichment analysis of cuproptosis-related genes.

Supplementary Table 3 The proportion of CNVs per cuproptosis-related genes in each cancer.

Supplementary Table 4 Association between the expression of cuproptosis-related genes and the tumor microenvironment in pan-cancer.

Supplementary Table 5 Association between the expression of cuproptosis-related genes and the infiltration levels of B cells, CD8+ T cells, CD4+ T cells, macrophages, neutrophils and dendritic cells in pan-cancer.

Supplementary Table 6 Association of the expression of cuproptosis-related genes with TMB, MSI, and tumor stemness (RNAss and DNAss) in pan-cancer.

Supplementary Figure 1 Heatmap demonstrating the expression profiles of cuproptosis-related genes in healthy tissues from the GTEx dataset.

Supplementary Figure 2 Boxplot demonstrating the expression levels of cuproptosis-related genes in different cancer types; data were extracted from the Cancer Cell Line Encyclopedia (CCLE).

Supplementary Figure 3 Differences in survival between the high- and low-methylation groups in each cancer. If the hazard ratio (HR) was >1, the high-methylation group was considered to have a higher risk of death; otherwise, the low- methylation group was considered to have a higher risk of death.

Supplementary Figure 4 The association between the expression of cuproptosis-related genes and immune infiltrate subtypes in all TCGA cancer types was analysed using ANOVA. C1 (wound healing); C2 (IFN-gamma dominant); C3 (inflammatory); C4 (lymphocyte depleted ); C5 (immunologically quiet); C6 (TGF-b dominant) (*P < 0.05; **P < 0.01; ***P < 0.001).
