# Supplementary figures and images for: Regulation, genomics, and clinical characteristics of cuproptosis regulators in pan-cancer
Source: Front Oncol. 2022 Oct 27;12:934076. doi: 10.3389/fonc.2022.934076 (PMC9647015; doi:10.3389/fonc.2022.934076)

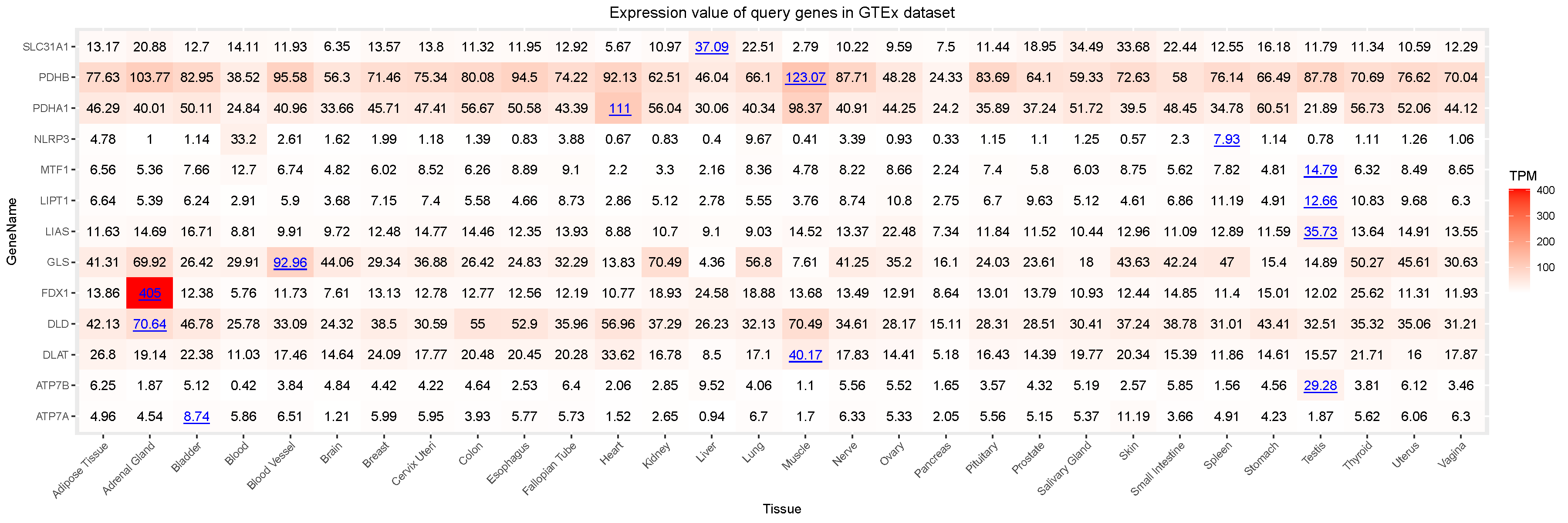

Supplement: Supplementary Figure 1 — Heatmap demonstrating the expression profiles of cuproptosis-related genes in healthy tissues from the GTEx dataset. [file Image_1.tif]

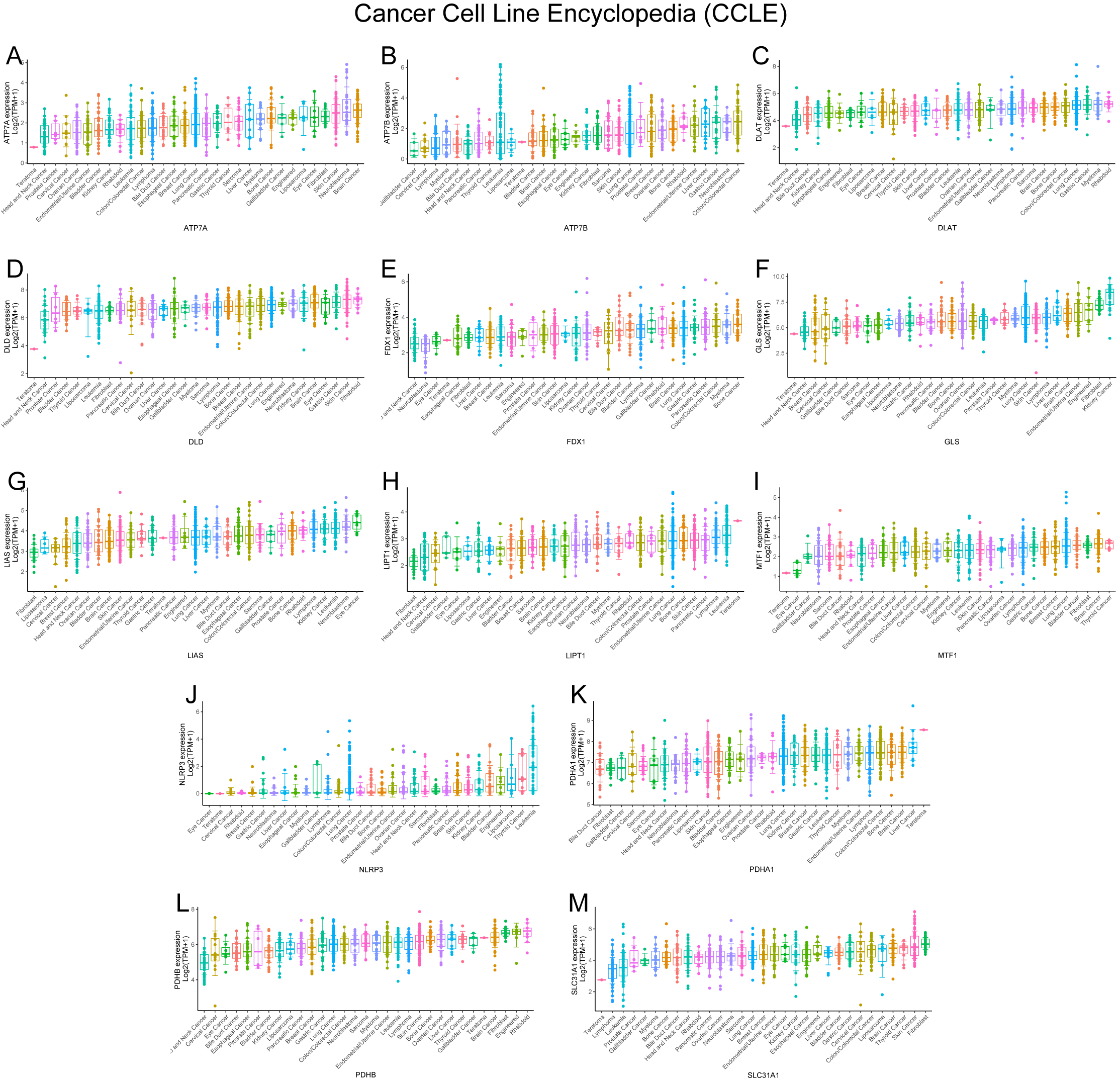

Supplement: Supplementary Figure 2 — Boxplot demonstrating the expression levels of cuproptosis-related genes in different cancer types; data were extracted from the Cancer Cell Line Encyclopedia (CCLE). [file Image_2.tif]

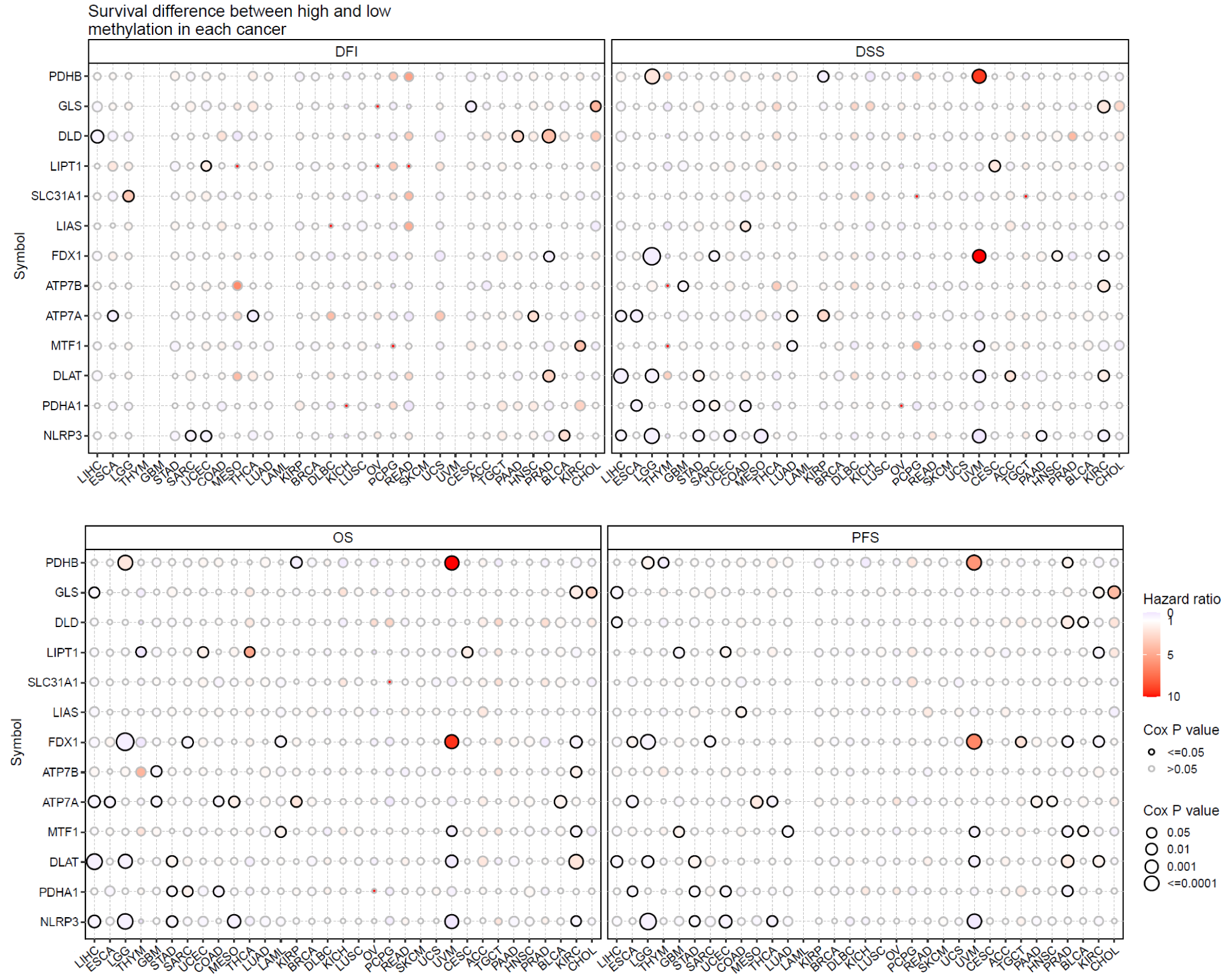

Supplement: Supplementary Figure 3 — Differences in survival between the high- and low-methylation groups in each cancer. If the hazard ratio (HR) was >1, the high-methylation group was considered to have a higher risk of death; otherwise, the low- methylation group was considered to have a higher risk of death. [file Image_3.tif]

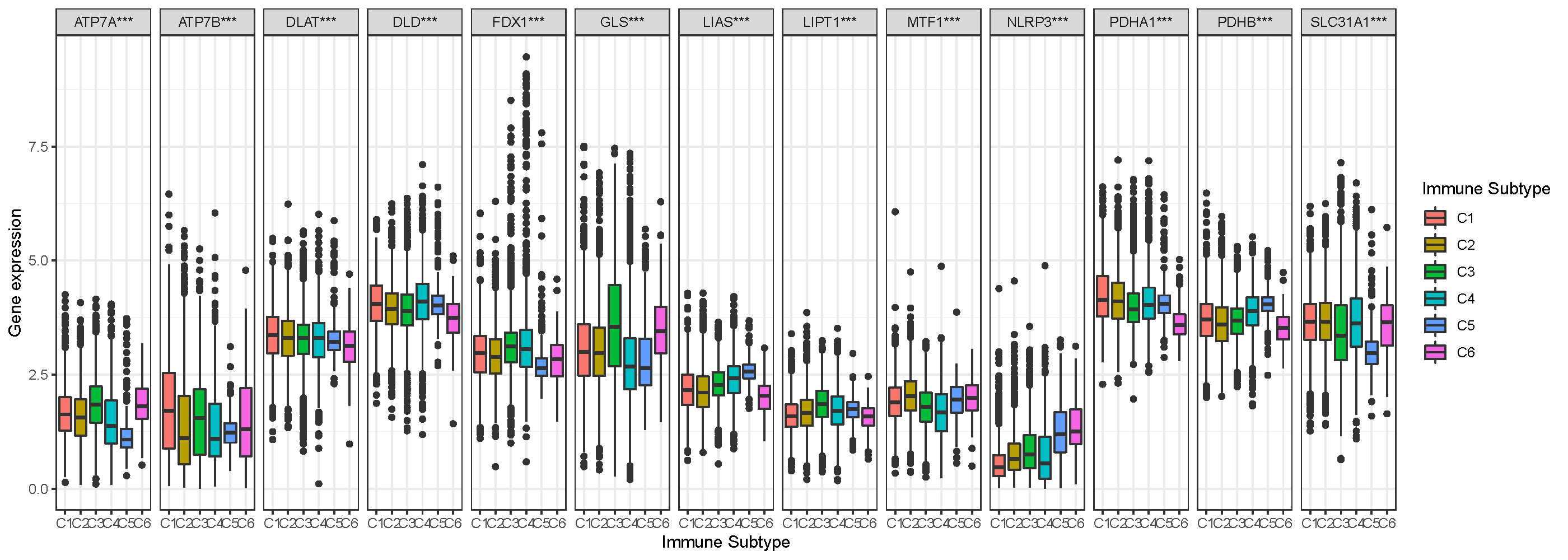

Supplement: Supplementary Figure 4 — The association between the expression of cuproptosis-related genes and immune infiltrate subtypes in all TCGA cancer types was analysed using ANOVA. C1 (wound healing); C2 (IFN-gamma dominant); C3 (inflammatory); C4 (lymphocyte depleted ); C5 (immunologically quiet); C6 (TGF-b dominant) (*P < 0.05; **P < 0.01; ***P < 0.001). [file Image_4.tif]
